# Orofacial skin inflammation increases the number of macrophages in the maxillary subregion of the rat trigeminal ganglion in a corticosteroid-reversible manner

**DOI:** 10.1007/s00441-020-03244-3

**Published:** 2020-07-21

**Authors:** Adam Legradi, Karolina Dulka, Gábor Jancsó, Karoly Gulya

**Affiliations:** 1 Department of Cell Biology and Molecular Medicine, University of Szeged, 4 Somogyi u, Szeged, H-6720 Hungary; 2 Department of Physiology, University of Szeged, 10 Dóm tér, Szeged, H-6720, Hungary

**Keywords:** Anthralin/dithranol, Iba1, Ki67, Macrophage, Trigeminal ganglion

## Abstract

Inflammation of the cutaneous orofacial tissue can lead to a prolonged alteration of neuronal and nonneuronal cellular functions in trigeminal nociceptive pathways. In this study, we investigated the effects of experimentally induced skin inflammation by dithranol (anthralin) on macrophage activation in the rat trigeminal ganglion. Tissue localization and protein expression levels of ionized calcium-binding adaptor molecule 1 (Iba1), a macrophage/microglia-specific marker, and proliferation/mitotic marker antigen identified by the monoclonal antibody Ki67 (Ki67), were quantitatively analyzed using immunohistochemistry and western blots in control, dithranol-treated, dithranol- and corticosteroid-treated, and corticosteroid-treated trigeminal ganglia. Chronic orofacial dithranol treatment elicited a strong pro-inflammatory effect in the ipsilateral trigeminal ganglion. Indeed, daily dithranol treatment of the orofacial skin for 3–5 days increased the number of macrophages and Iba1 protein expression in the maxillary subregion of the ipsilateral ganglion. In the affected ganglia, none of the Iba1-positive cells expressed Ki67. This absence of mitotically active cells suggested that the accumulation of macrophages in the ganglion was not the result of resident microglia proliferation but rather the extravasation of hematogenous monocytes from the periphery. Subsequently, when a 5-day-long anti-inflammatory corticosteroid therapy was employed on the previously dithranol-treated orofacial skin, Iba1 immunoreactivity was substantially reduced in the ipsilateral ganglion. Collectively, our findings indicate that both peripheral inflammation and subsequent anti-inflammatory therapy affect macrophage activity and thus interfere with the functioning of the affected sensory ganglion neurons.

## Introduction

Microglial cells belong to the monocyte/macrophage lineage (Kreutzberg 1996; Prinz et al. 2011) and constitute an ontogenetically distinct population in the mononuclear phagocyte system (Ginhoux et al. 2010). In nervous tissues, microglia are ubiquitous as a continuum of multiple phenotypes, including ameboid and extremely ramified morphologies. Under physiological conditions, microglia display a ramified morphology with subdued macrophage-like functional properties. However, in response to neural injuries, infections, or inflammation, microglia become activated, displaying a number of characteristic morphological, molecular, immunological, and functional changes (Kreutzberg 1996; Kettenmann et al. 2011; Salter and Beggs 2014). This transformation parallels microglial proliferation, and homing and adhesion to damaged cells (Streit et al. 1999). In addition, this process is known to be coupled to vigorous phagocytosis and phenotypic conversion (Szabo and Gulya 2013). Indeed, the ameboid appearance and the phagocytic nature of microglia coincide with their antigen presentation capability and cytotoxic and inflammation-mediating signalization (Town et al. 2005). In tissues, microglia respond to both pro- and anti-inflammatory signals and concomitantly produce pro- and anti-inflammatory factors (Kata et al. 2016, 2017).

Dithranol (anthralin; 1,8-dihydroxy-9(10H)-anthracenone) is widely used as an effective topical treatment for patients with psoriasis (Hendriks et al. 2012). The precise molecular basis for dithranol’s actions is believed to be related to the production of free radicals and the keratinization of the epidermis, along with its strong irritative and pro-inflammatory properties (Sehgal et al. 2014). However, orofacial tissue inflammation can result in prolonged neuronal activation in trigeminal nociceptive pathways. We previously reported that chronic orofacial dithranol treatment results in a complete loss of the pan-neuronal marker protein ubiquitin carboxyl-terminal hydrolase L1 (UCH-L1, also known as PGP 9.5) immunopositivity after 5 days (Orojan et al. 2006). In subsequent experiments, we demonstrated that dithranol treatment transsynaptically downregulates the gene expression of the intracellular calcium-binding protein calmodulin (CaM) in rat principal sensory and motor trigeminal nuclei, in which altered amounts of CaM mRNA were observed in response to the treatment (Orojan et al. 2008). Aside from changes in neurochemical markers, experimental manipulations of the infraorbital branch of the trigeminal nerve (Xu et al. 2008) and inflammation of the temporomandibular joint (Villa et al. 2010) have been shown to cause a persistent increase in the number of glial fibrillary acidic protein-positive satellite cells in the trigeminal ganglion.

In this study, we investigated whether the induction of chronic orofacial inflammation by dithranol or a subsequent corticosteroid treatment resulted in an altered macrophage activity in the trigeminal ganglion. Our findings draw attention to the possibility that chronic peripheral inflammation, or anti-inflammatory treatment, could affect neuronal functioning in the trigeminal ganglion via macrophage activation.

## Materials and methods

### Animal handling and treatments

Experimental procedures were carried out in strict compliance with the European Communities Council Directive (86/609/EEC) and followed the Hungarian legislation requirements (XXVIII/1998 and 243/1998) and university guidelines regarding the care and use of laboratory animals. All experimental protocols were approved by the Institutional Animal Welfare Committee of the University of Szeged (I-74-II/2009/MÁB). Adult (200–220 g) male Sprague Dawley rats were maintained under standard housing conditions and kept on a normal diet with tap water ad libitum and a 12-h light cycle. The infraorbital region of the orofacial skin (about 1 cm^2^ around the whisker pad) on the right side of the animal was treated daily with a Vaseline-based paste (petrolatum or vaselinum album; Sigma, St. Louis, MO, USA) containing 10% dithranol (Sigma) for 3 or 5 days (Orojan et al. 2006, 2008). Approximately 90 and 150 mg of dithranol-containing paste were applied for 3 and 5 days, respectively, to the surface of the treated skin. The contralateral side of the animals received only Vaseline paste and served as controls. After the fifth day of dithranol treatment, one group of animals was ipsilaterally treated daily for 5 days with a 0.1% corticosteroid lotion (Elocon®; mometasone furoate, 9α,21-dichloro-11β,17α-dihydroxy-16α-methylpregna-1,4-diene-3,20-dione 17α-(2-furoate); Schering, Kenilworth, NJ, USA). Approximately 400 mg of mometasone furoate lotion was applied at each time to the surface of the treated skin (Orojan et al. 2006, 2008). Dithranol-treated animals began to rub their perioral area, predominantly with their ipsilateral paw (data not shown), within 1 h and continued to do so throughout the entire treatment regimen. Reddened perioral swelling was evident on the treated infraorbital region after about 3 h. Rats receiving the vehicle did not exhibit paw-related behavioral reactions or perioral inflammation. Rats were decapitated on the day following the last treatment.

### Histology

**Fig. 1:**
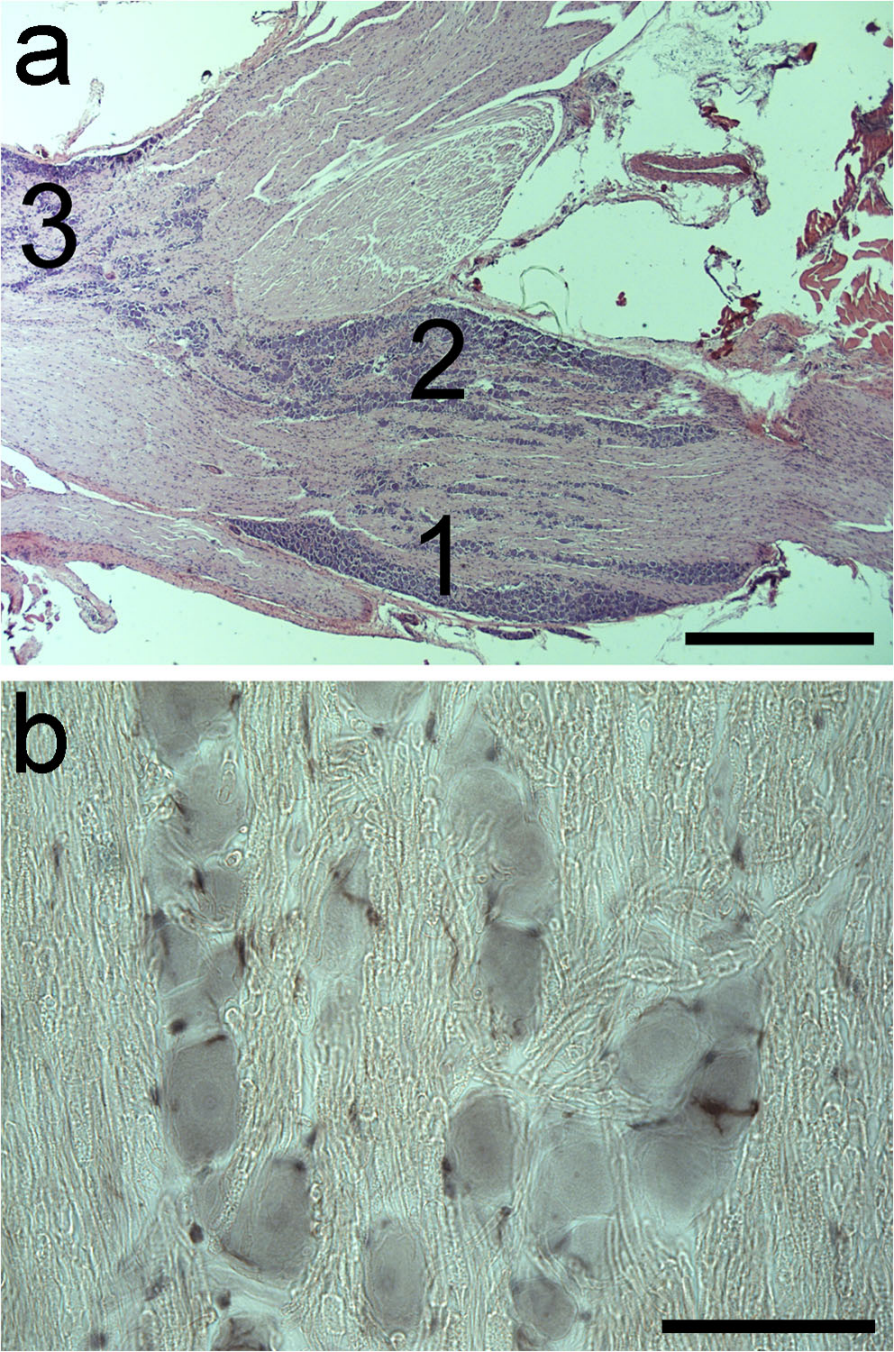
Cytoarchitecture of the rat trigeminal ganglion. (a) Localization of the three neuron-containing subdivisions of the trigeminal ganglion in an H&E-stained, paraffin-embedded tissue section of a control animal. (1) Ophthalmic neurons, (2) maxillary neurons, and (3) mandibular neurons (nomenclature adapted from Xu et al. (2008) and Waite and Tracey (1995)). A quantitative microscopic analysis was performed on the maxillary subregion of the trigeminal ganglion. Scale bar, 1 mm. (b) Localization of Iba1-immunopositive macrophages around the ganglionic cells in a 20 μm thick cryostat section from the maxillary subdivision of the ganglion from a control rat. The majority of the macrophages surround the ganglionic cells, whereas others are located within the nerve fibers. Scale bar, 75 μm

Following the different dithranol and/or corticosteroid treatments, animals were transcardially perfused with 4% formaldehyde in 0.05 M phosphate-buffered saline (PBS). The trigeminal ganglia were quickly removed from the animals and used for cryostat sectioning or embedded in paraffin for hematoxylin and eosin (H&E) staining and immunohistochemistry. Tissues were serially sectioned using a cryostat (20 μm) on gelatin-coated glass slides and kept at − 20 °C until further processing for histology and immunohistochemistry (up to 2 or 3 days). Paraffin-embedded sections were cut (6 μm) on a microtome (Leica RM2235; Leica Mikrosysteme Vertrieb GmbH, Wetzlar, Germany) and mounted on slides coated with (3-aminopropyl)triethoxysilane (Menzel, Darmstadt, Germany) and used for histology and immunohistochemistry. Quantitative analyses of ionized calcium-binding adaptor molecule 1 (Iba1)–positive macrophages and eosin-stained neurons were performed in the maxillary subregion of the contra- and ipsilateral ganglia (Fig. 1(a)), which is where the cell bodies of most of the afferent fibers that innervate the orofacial skin, upper lip, mystacial vibrissae, lateral nose, and upper teeth are located (Waite and Tracey 1995).

### Antibodies

For localization and quantitative analyses of macrophage phenotypes in the trigeminal ganglia, an antibody against the intracellular actin- and Ca^2+^-binding protein, Iba1 (Szabo and Gulya 2013), was used for immunohistochemistry and western blotting. An anti-proliferation marker antigen identified by the monoclonal antibody Ki67 (anti-Ki67) was used to detect proliferating cells. Ki67 is a nuclear protein expressed in all active phases of the cell cycle, from the late G1 phase through the end of the M phase, but is absent in nonproliferating and early G1 phase cells (Szabo et al. 2016; Cuylen et al. 2016). The neuronal marker, the anti-NeuN antigen, was used to identify ganglion cells (Beliczai et al. 2008). The anti-glyceraldehyde 3-phosphate dehydrogenase (GAPDH) antibody was used as an internal control in western blot experiments (Wu et al. 2012). Appropriate dilutions of primary and secondary antibodies, along with incubation times and blocking conditions, were carefully determined for both immunohistochemistry and western blot analyses. In order to detect the specificity of the secondary antibodies, omission control experiments (i.e., staining without the primary antibody) were performed. In these experiments, no immunohistochemical signals were observed.

### Immunohistochemistry

Immunohistochemistry was performed as previously described (Szabo and Gulya 2013). For light microscopic immunohistochemistry, the endogenous peroxidase activity of the cryostat sections was blocked with 1% H_2_O_2_ in 0.05 M PBS for 10 min at 37 °C. After washing in 0.05 M PBS three times for 5 min, the samples were blocked and permeabilized in a 0.05 M PBS solution containing 0.1% Triton X-100, 5% normal goat serum (NGS), and 10% bovine serum albumin for 30 min at 37 °C. Sections were then incubated with a polyclonal rabbit antibody against Iba1 (final dilution 1:300; Wako, Osaka, Japan) in the above solution at 4 °C overnight. Sections were then washed several times in 0.05 M PBS, followed by incubation with a biotinylated anti-rabbit immunoglobulin G (IgG) secondary antibody (final dilution 1:200; Amersham Biosciences, Little Chalfont, Buckinghamshire, UK) for 6 h at room temperature (RT). After several washes in 0.05 M PBS at RT, sections were incubated with a biotinylated streptavidin peroxidase tertiary antibody (final dilution 1:200; Amersham) at 4 °C overnight. Immunoreactivity was visualized with the SigmaFast DAB tablet system (Sigma) according to the manufacturer’s instructions. The sections were then dipped in distilled water, stained with 0.5% eosin solution for 30 s, dehydrated with ethanol, covered with a DPX mounting medium (Fluka, Buchs, Switzerland), and coverslipped. Representative microphotographs from the trigeminal ganglia were taken with a digital microscope camera (QImaging MicroPublisher 3.3 RTV; QImaging Inc., Surrey, BC, Canada) attached to a Leica DMLB microscope (Leica). Image processing was performed using the QCapture Pro 6.2 software for Windows (QImaging Inc.). The areas of ganglion cells and macrophages were counted from five fields of view in four sections for each animal (*n* = 4–6 rats in each treatment group) using a × 40 objective. The investigator was blind to the treatments of the samples.

For fluorescent immunohistochemistry, paraffin-embedded tissue sections were deparaffinized, rehydrated, and placed in a jar filled with 0.01 M citrate buffer (pH 6.0) containing 0.05% Tween 20 and heated at 95 °C for 20 min. The sections were washed 3 × 10 min in 0.05 M PBS containing 0.05% Tween 20 and blocked in 0.05 M PBS solution containing 0.05% Tween 20 and 5% NGS for 1 h at RT. The sections were then incubated with rabbit polyclonal anti-Iba1 (final dilution 1:300; Wako) antibody, mouse monoclonal anti-NeuN antibody (1:100 final dilution; Chemicon, Temecula, CA, USA), or mouse monoclonal anti-Ki67 IgG (final dilution 1:500; Cell Signaling Technology, Danvers, MA, USA) in a 0.05 M PBS solution containing 0.05% Tween 20 and 5% NGS overnight at 4 °C. After washing (4 × 10 min in 0.05 M PBS containing 0.05% Tween 20), primary antibodies were labeled fluorescently with either Alexa 568–conjugated anti-rabbit IgG or Alexa 488–conjugated anti-mouse IgG secondary antibody (final dilution for both 1:1000; Invitrogen, Carlsbad, CA, USA) in a blocking solution for 3 h at RT. After 4 × 10 min washes in 0.05 M PBS containing 0.05% Tween 20, cell nuclei were stained in 0.05 M PBS containing 1 mg/mL polyvinylpyrrolidine and 0.5 μL/mL Hoechst 33258 dye (Invitrogen). Sections were finally washed 2 × 5 min in 0.05 M PBS containing 1 mg/mL polyvinylpyrrolidine, covered with VECTASHIELD (Vector Laboratories, Peterborough, UK), and coverslipped. The sections were then viewed on a Nikon Microphot-FXA epifluorescence microscope (Nikon Corp., Tokyo, Japan) and photographed with a Spot RT Color CCD camera (SPOT RT/KE; Diagnostic Instruments, Inc., Sterling Heights, MI, USA).

### Western blot analysis

The trigeminal ganglia were removed and individually homogenized in 50 mM Tris-HCl (pH 7.5 at 4 °C) containing 150 mM NaCl, 0.1% Nonidet P-40, 0.1% cholic acid, 2 μg/mL leupeptin, 1 μg/mL pepstatin, 2 mM phenylmethylsulfonyl fluoride, and 2 mM EDTA and centrifuged at 14,000×*g* for 10 min at 4 °C. The pellets were discarded and the protein concentrations from the supernatants were determined (Lowry et al. 1951). Western blot analysis was performed as previously described (Szabo and Gulya 2013). Equal amounts of proteins in the linear range of detection on a polyacrylamide gel (Aldridge et al. 2008) were loaded. Briefly, protein samples (25 μg) denatured at 100 °C for 5 min were loaded to wells and separated on 12% sodium dodecyl sulfate (SDS)–polyacrylamide gels and transferred onto Hybond ECL nitrocellulose membranes (Amersham Biosciences), blocked in 5% nonfat dry milk in 0.1 M Tris-buffered saline (TBS) containing 0.1% Tween 20 for 1 h, and then incubated with either a rabbit anti-Iba1 polyclonal antibody (dilution 1:1000; Wako) or a mouse anti-GAPDH monoclonal antibody (clone GAPDH-71.1; 1:20,000 final dilution; Sigma). Nonspecifically bound or excess antibody was removed with 5 × 5 min rinses in 0.1 M TBS containing 0.1% Tween 20. Membranes were then incubated for 1 h with either peroxidase-conjugated goat anti-rabbit or peroxidase-conjugated rabbit anti-mouse antibody (dilution 1:2000; Jackson ImmunoResearch Europe Ltd., Cambridgeshire, UK) and washed three times. The enhanced chemiluminescence method (ECL plus western blotting detection reagents; Amersham) was used to reveal immunoreactive bands according to the manufacturer’s instructions.

### Image and statistical analyses

Digital microscopic images were acquired with a Nikon Microphot-FXA epifluorescence microscope (Nikon Corp.) using a Spot RT Color CCD camera and Spot RT software (Spot RT/KE; Diagnostic Instruments, Inc.). Cross-sectional areas of Iba1 immunopositive macrophages and eosin-stained neurons were measured in the pictures taken from the maxillary infraorbital region of the trigeminal ganglia (Fig. 1 (a) and (b)). Digital image production was performed with the Adobe Photoshop CS5.1 software (Adobe Systems, Inc., San Jose, CA, USA). Color correction (brightness, contrast) and cropping of the fluorescent images were occasionally performed when individual photomicrographs were assembled as figure panels for publication.

**Fig. 2:**
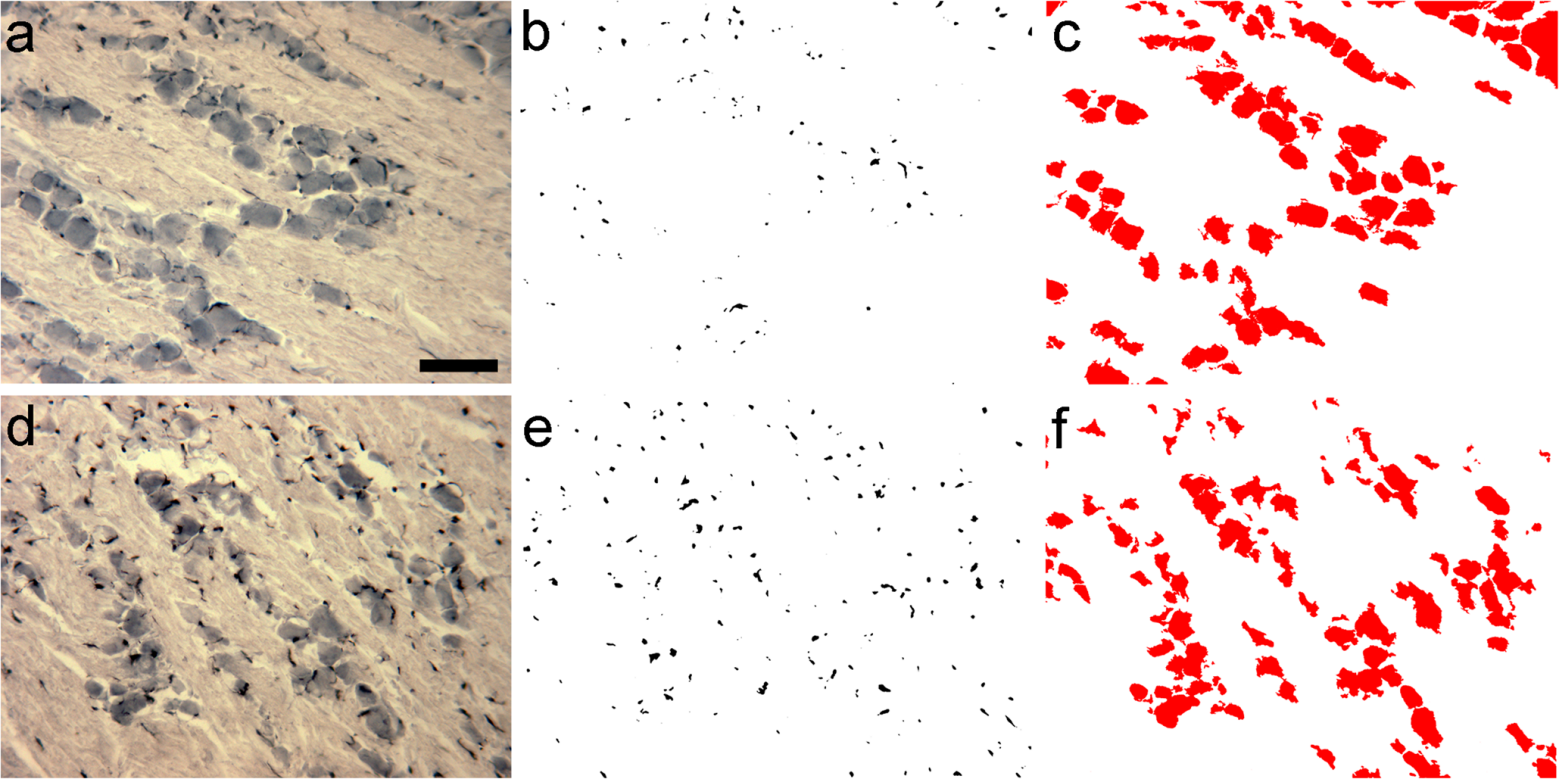
Quantitation of macrophage area/neuron area in the maxillary subdivision of the trigeminal ganglion. Iba1 immunohistochemistry and quantitative data from the maxillary subregions of ganglia obtained from control (a, b, c) and dithranol-treated (d, e, f) animals are shown. Paraffin-embedded tissue sections were processed for Iba1 immunohistochemistry and counterstained with eosin. Cross-sectional areas of macrophages and neurons were identified and measured in digital images as described in “Materials and methods.” (a), (d) Iba1 immunohistochemistry; (b), (e) binary silhouettes of Iba1-labeled cells (c), (f) binary silhouettes of eosin-counterstained neurons. Scale bar in (a), 100 μm

Paraffin-embedded tissue sections (6 μm) of the trigeminal ganglia were deparaffinized, rehydrated, and processed for Iba1 immunohistochemistry (Fig. 2(a), (d)). The sections were counterstained with eosin, dehydrated, and covered with DPX mounting medium. Iba1 immunoreactive cell images or eosin-stained neurons were converted into binary replicas by using threshold procedures implemented by Photoshop and ImageJ (version 1.47; developed at the US National Institutes of Health by W. Rasband, available at https://imagej.net/Downloads; Schneider et al. 2012) as we published earlier (Szabo and Gulya 2013; Kata et al. 2016; Szabó et al., 2016). Briefly, digital images in tagged image file formats (.tif) were opened in Photoshop; then, threshold was set in the Adjustment menu until silhouettes overlapped Iba1-labeled cells and processes (Fig. 2(b), (e)) or the eosin-counterstained ganglion cells (Fig. 2(c), (f)). Areas of macrophages and neurons were measured with the use of the computer program ImageJ. Saved images were opened in ImageJ, where image type was set to 8 bit and the Process menu was selected to make binary silhouettes. The Set Scale menu was then selected, and the following parameters were set: distance in pixel, 420; known distance, 75; pixel aspect ratio, 1.0; unit of length, μm. Analyze particles menu was selected, where size and circularity were set to 0-infinity and 0-1, respectively. Displayed results were copied to Excel (Microsoft Corp., Redmond, WA, USA) and analyzed. Twenty fields in four sections for every treatment group were statistically analyzed with one-way analysis of variance (ANOVA) using SigmaPlot v.12.3 (Systat Software, Inc., San Jose, CA, USA).

Grayscale digital images of the western blots were acquired by scanning the autoradiographic films with a desktop scanner (Epson Perfection V750 Pro; Seiko Epson Corp., Nagano, Japan). Images were scanned and processed at identical settings to allow comparisons of the western blot results from different samples. The immunoreactive densities of lanes equally loaded with protein were quantified, and all samples were normalized to the internal GAPDH load controls as we previously reported (Kata et al. 2016, 2017). For western blotting, one-way ANOVA (SigmaPlot) or Student’s *t* test was used for statistical comparisons. Values are presented as the mean ± standard error of the mean (SEM) from at least four immunoblots, each representing an independent experiment. A *p* value of < 0.05 was considered significant; * denotes *p* < 0.05.

## Results

**Fig. 3:**
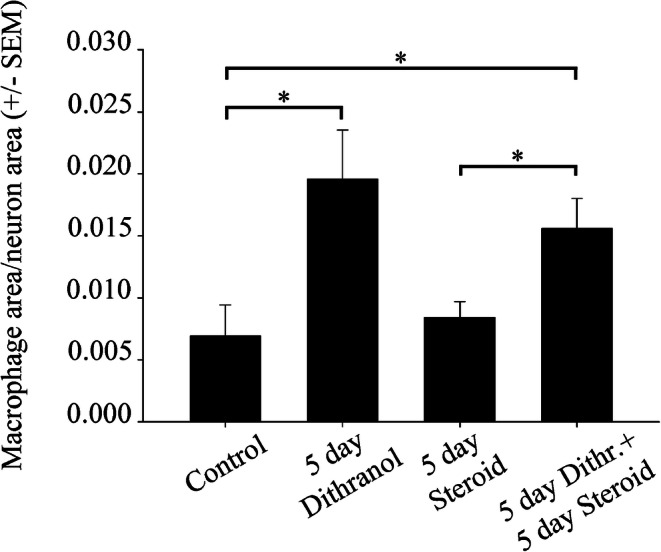
The macrophage area/neuron area ratio increases after dithranol treatment in the maxillary subdivision of the trigeminal ganglion. The orofacial skin was treated with dithranol for 5 days, dithranol for 5 days followed by corticosteroid for 5 days, and corticosteroid for 5 days. A quantitative analysis of the macrophage/neuron ratio in the maxillary subregion of the trigeminal ganglion was carried out by computing the cross-sectional areas of Iba1-immunopositive macrophages and eosin-counterstained ganglion cells on tissue sections from controls and treated animals. A 5-day-long dithranol treatment significantly increased the macrophage area/neuron area ratio (*p < 0.05; one-way ANOVA with Dunn’s post hoc test) in the maxillary subregion. This ratio decreased, albeit non-significantly, in dithranol + steroid-treated ganglia. Steroid treatment alone was without effect on this ratio. Area measurements were made in 4 non-overlapping but adjacent fields of view from four nonconsecutive sections for each animal (*n* = 4–6 rats in each experimental group). Data are presented as the mean ± SEM of the ratio of Iba1-positive cell areas to eosin-stained ganglionic cell areas

The three subregions (ophthalmic, maxillary, mandibular) of the trigeminal ganglion are shown in Fig. 1(a). In control rats, only few Iba1-immunopositive macrophages surround neurons in the maxillary subregion (Figs. 1(b) and 2(a)). Daily dithranol treatment to the infraorbital region of the orofacial area for 3 and 5 days caused severe skin inflammation (for a histological evaluation, see Orojan et al. 2006). Light microscopic immunohistochemistry revealed that 5-day-long dithranol treatment increased the amounts of Iba1-immunoreactive components (Fig. 2(d), (e)) in the maxillary subdivision of the ganglion over the control samples (Fig. 2(a), (b)). Quantitative immunohistochemical analysis revealed that the macrophage area/neuron area ratio in the maxillary subregion was significantly increased (*p < 0.05) after 5 days of dithranol treatment as compared with controls (Fig. 3). Steroid treatment alone did not affect the macrophage area/neuron area ratios. When the dithranol treatment was followed by a 5-day-long corticosteroid treatment to the same orofacial skin region, the macrophage area/neuron area ratio was lowered, albeit non-significantly, in the maxillary subregion of the ganglia.

**Fig. 4:**
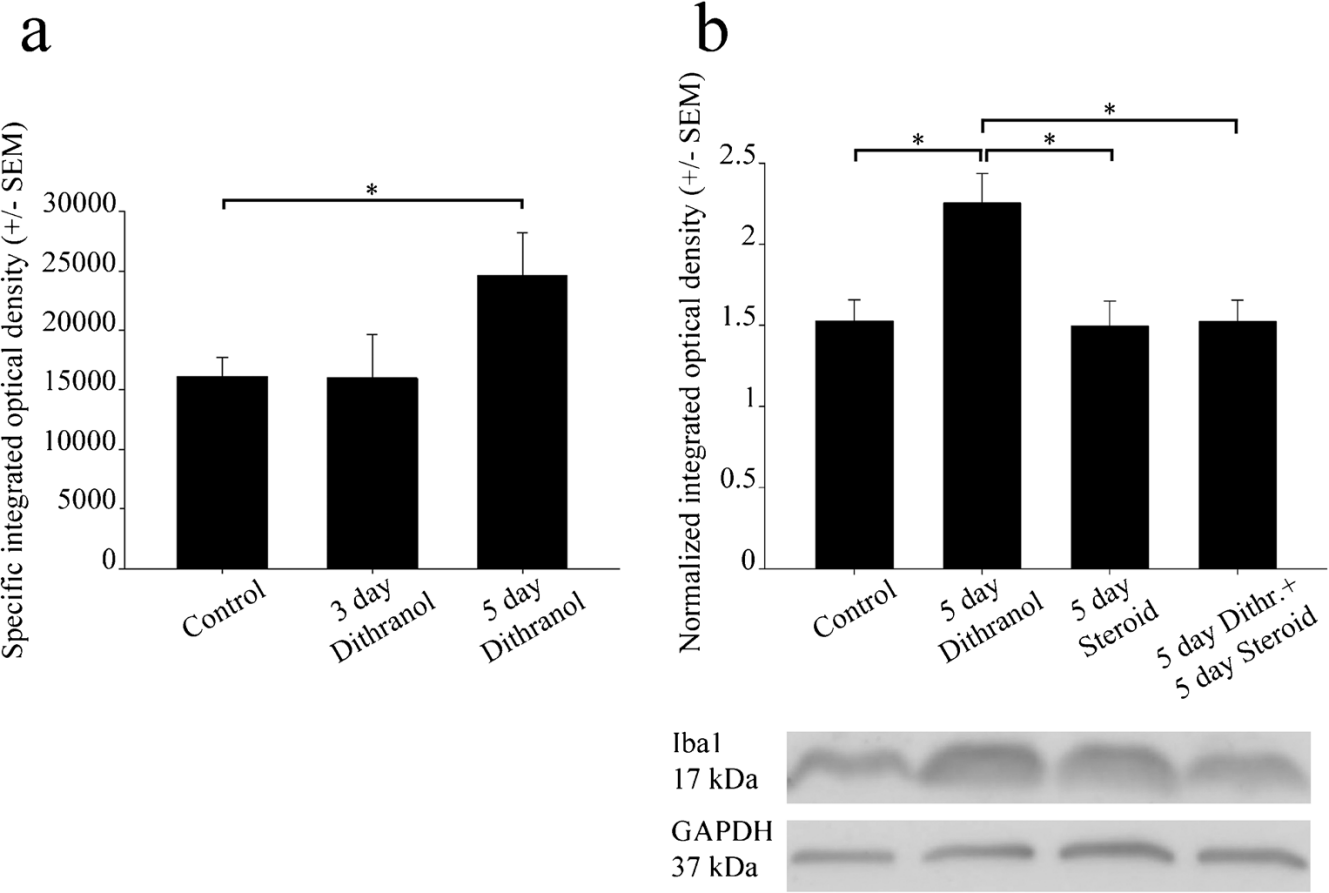
Orofacial tissue inflammation and subsequent corticosteroid treatment antagonistically regulate Iba1 protein levels in the trigeminal ganglia. Whole ganglia were used in this experiment. Contra- (control) and ipsilateral (treated) ganglia were separately homogenized, and 25 μg of protein was subjected to SDS-polyacrylamide gel electrophoresis. Protein bands were transferred to nitrocellulose membranes and assayed for reactivity toward a polyclonal rabbit antibody directed against the Iba1 protein. (a) Iba1 immunoreactivity was altered dose-dependently in the ipsilateral ganglia. After 5 days of dithranol treatment, a significant increase in Iba1 immunopositivity was detected (*p < 0.05) in the ipsilateral trigeminal ganglion as compared with controls (24,611 ± 3628 and 16,142 ± 1580 specific integrated optical density values, respectively). This effect was dose-dependent, as only the larger dose of dithranol was effective. (b) Corticosteroid antagonized the effect of dithranol with regard to the Iba1 content of the trigeminal ganglion, as demonstrated by integrated optical density values normalized to the internal GAPDH load controls. A chronic, 5-day-long dithranol treatment regimen significantly elevated this immunoreactivity, whereas a subsequent 5-day-long corticosteroid treatment regimen significantly depleted this immunoreactivity (*p < 0.05; one-way ANOVA with Tukey’s post hoc test)

Western blot analyses confirmed that the effect of dithranol was dose-dependent, as only the 5-day-long dithranol treatment increased Iba1 immunoreactivity significantly in the affected ganglion (Fig. 4(a)). In addition, when corticosteroid was applied to the dithranol-treated orofacial area for 5 days, a significant decrease in Iba1 immunoreactivity was observed in the ipsilateral trigeminal ganglion by western blot analysis (Fig. 4(b)). Corticosteroid treatment alone for 5 days was ineffective as it did not change the immunoreactive signal of the blot significantly as compared with the controls.

**Fig. 5:**
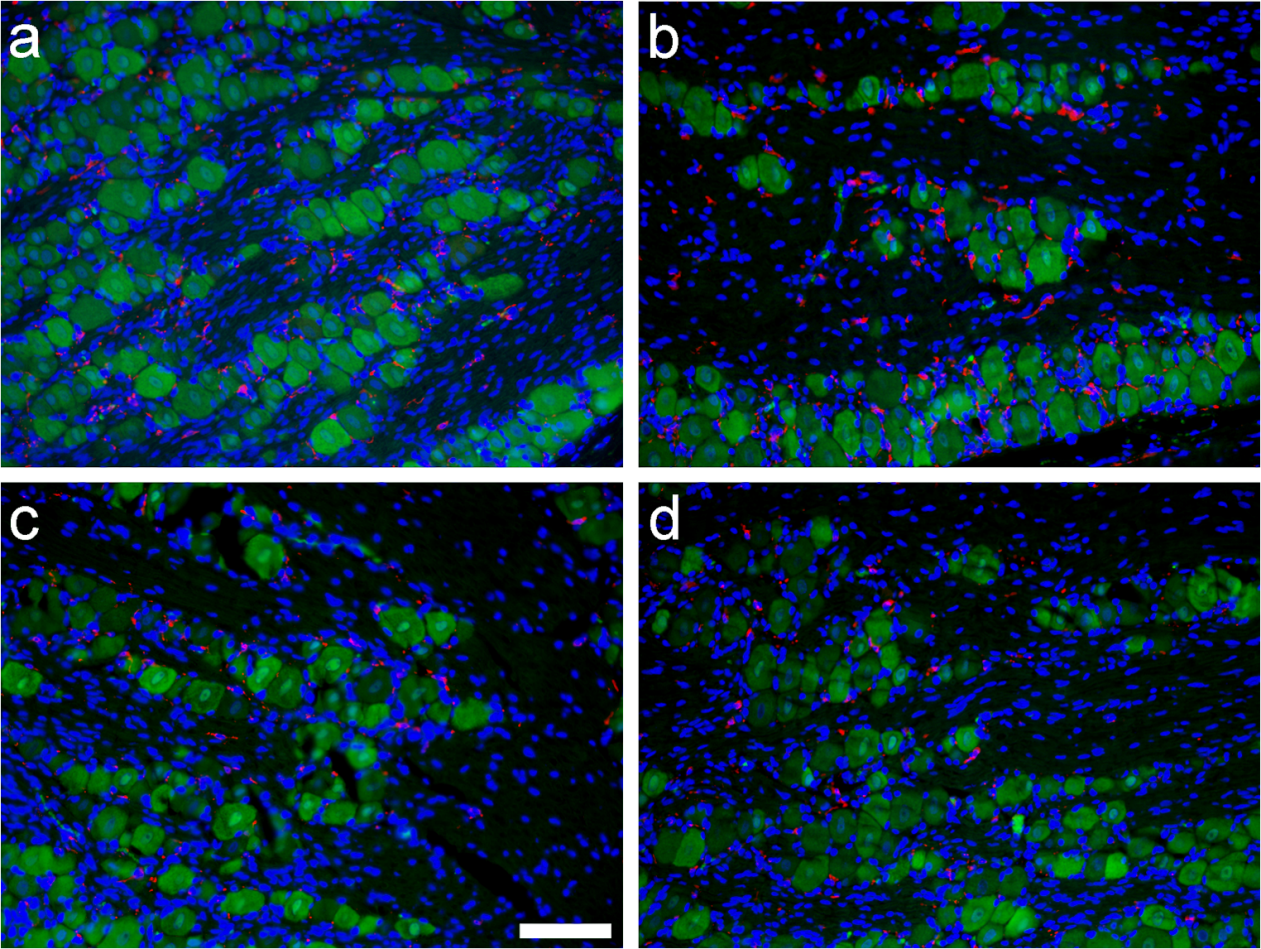
Orofacial tissue inflammation increases, while subsequent corticosteroid treatment decreases, Iba1 immunoreactivity in the maxillary subdivision of the trigeminal ganglion. After a 5-day-long dithranol treatment regimen, rats were transcardially perfused and the contra- and ipsilateral trigeminal ganglia were removed, embedded in paraffin, and sectioned. In these composite images, Iba1- and NeuN-expressing macrophages (red) and neurons (green), respectively, were detected by double fluorescence immunohistochemistry. Cell nuclei (blue) were stained with Hoechst 33258 dye. (a) Iba1 and NeuN immunoreactivities in the maxillary subregion of the contralateral (control) trigeminal ganglion after sham treatment. (b) Iba1 immunoreactivity increased after a chronic, 5-day-long dithranol treatment regimen in the maxillary subregion of the ipsilateral trigeminal ganglion. (c) Corticosteroid treatment alone did not have any effect on Iba1 immunoreactivity as compared with the contralateral (control) trigeminal ganglion. (d) When the dithranol treatment was followed by a 5-day-long corticosteroid treatment on the same orofacial skin area, a decrease in Iba1 immunoreactivity was detected. Scale bar, 100 μm

**Fig. 6:**
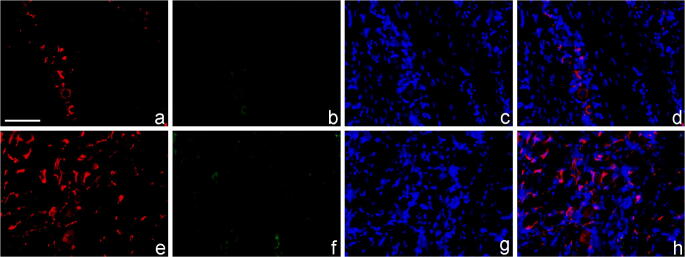
The number of Iba1-immunopositive cells increases in the maxillary subdivision of the trigeminal ganglion without an increase in the number of mitotic cells. The vast majority of Iba1-expressing cells in the maxillary subregion of the trigeminal ganglion were devoid of Ki67 immunoreactivity after dithranol treatment, indicating the absence of mitotic cells. The number of Iba1-expressing cells (red) increased after dithranol treatment in the ipsilateral ganglion (e) as compared with the contralateral side (a). The absence of Ki67-labeled mitotic cells (green) (b, f) suggested that the many Iba1-positive cells detected after dithranol treatment were hematogenous macrophages that migrated recently to the ganglion (f). Cell nuclei (blue) were stained with Hoechst 33258 dye (c, g). Merged pictures are shown (d, h). Scale bar, 100 μm

Double immunofluorescence histochemistry (Fig. 5) corroborated the results of the western blotting, as the Iba1-positive macrophage profiles (sections of somata and processes) increased after dithranol treatment (Fig. 5(b)). In order to examine whether the increased number of Iba1-expressing cells in the ipsilateral trigeminal ganglia after dithranol treatment was the result of resident macrophage proliferation, double immunofluorescent histochemistry was performed using both Iba1 and Ki67 antibodies. Condensed chromatin in M phase and/or Ki67 immunoreactivity from late G1 to late M phase (both are markers of cellular proliferation/mitotic activity) in Iba1-expressing macrophages was not observed in the ganglion (Fig. 6 (b) and (f)). Thus, Iba1-positive cells in the ganglion likely represented recently migrated macrophages into the maxillary subregion of the ganglion rather than proliferating resident macrophages.

## Discussion

The innervation territories of the three main divisions of the trigeminal nerve in rodents are well established (Waite and Tracey 1995). In the rat, the maxillary division of the trigeminal nerve innervates infraorbital skin around the whisker pad, providing both deep and superficial nerves to the vibrissal follicles. Experimentally induced lesions (Takahashi et al. 2011; Xu et al. 2008) and pharmacological interventions (Cady et al. 2010) targeting the trigeminal nerve result in neuroanatomical and chemical alterations of both the neuronal and the glial components of the trigeminal ganglion. For example, herpes simplex virus infection (Mori et al. 2003) or lipopolysaccharide-induced systemic inflammation (Franceschini et al. 2013) increases the number of Iba1-immunoreactive macrophages in the ganglion. Iba1 is a calcium-binding protein (Ahmed et al. 2007) that is expressed not only in the microglia of the central nervous system (CNS) but also in the macrophages of the peripheral nervous system (PNS), including the trigeminal ganglion (Glenn et al. 1993).

In a previous study, we demonstrated that chronic dithranol treatment causes severe, but corticosteroid-reversible, inflammation in the infraorbital region of the orofacial skin, which results in a complete loss of the pan-neuronal marker UCHL-1 (also known as PGP9.5) in the nerve fibers of the affected skin area (Orojan et al. 2006). The action of dithranol is believed to be mediated by the generation of reactive oxygen species and anthrone radicals that ignite a series of pro-inflammatory pathways that can eventually destroy nerve endings (Orojan et al. 2006). Damage to peripheral nerves results in marked microgliosis in the somatotopically relating central projection territories of the affected nerve (Gilmore and Skinner 1979; Beggs and Salter 2007). Similarly, inflammatory processes in the trigeminal region, for example, in the temporomandibular joint, elicit activation of microglia in both the spinal trigeminal nucleus and the trigeminal ganglion (Villa et al. 2010). In the present study, we demonstrated a marked increase in the total cross-sectional area of Iba1-immunoreactive macrophages in the maxillary subregion of the trigeminal ganglion following a dithranol-induced cutaneous inflammatory reaction in the orofacial region of the rat. Activated microglia produce pro-inflammatory cytokines (Streit et al. 1998; Takahashi et al. 2011) and increase the synthesis of Iba1 (Szabo and Gulya 2013). Consistent with previous in vivo (Endo et al. 2014; Kiefer and Kreutzberg 1991) and in vitro (Hinkerohe et al. 2010) results, corticosteroid treatment administered alone to the ipsilateral orofacial skin did not affect macrophage proliferation or Iba1 production in the trigeminal ganglion but was able to reduce the effects of dithranol-induced inflammation.

It can be debated whether this response is due to the local proliferation of Iba1-positive cells or an increased influx of macrophages from the blood. Indeed, during inflammation, one of the most important processes is leukocyte extravasation (Lange et al. 1998). In addition, there may be differences in microglia/macrophage activation and proliferation between the CNS and PNS as a consequence of harmful stimuli (Leskovar et al. 2000; Greenhalgh and David 2014). However, it is difficult to identify hematogenous macrophages and distinguish them from resident tissue macrophages, as they both share the same marker proteins, such as Iba1, CD68, and CX3C chemokine receptor 1 (CX3CR1). These cell types can be crudely distinguished on the basis of differing levels of cluster of differentiation 45 (CD45) or C-C chemokine receptor type 2 (CCR2) proteins (David and Kroner 2011) and their differential susceptibility to radiation (Matsumoto and Fujiwara 1987). Nonetheless, their discrimination becomes even more difficult as infiltrating macrophages quickly become indistinguishable from resident macrophages.

Experiments using bone marrow chimeras with lethally irradiated recipients have been conducted in order to overcome this issue. It has been concluded in many of these studies that bone marrow–derived monocytes can cross the blood–brain barrier (BBB) and differentiate into parenchymal microglia of the brain or spinal cord (Djukic et al. 2006; Echeverry et al. 2011; Sawada et al. 2014; Zhang et al. 2007). For example, Menasria et al. (2015) provided evidence that blood monocyte–derived macrophages infiltrate the brain in experimental herpes simplex virus 1 encephalitis and the infiltrating cells from the monocytic lineage differentiate into activated, Iba1-expressing resident tissue macrophages. However, Schilling et al. (2003) demonstrated using bone marrow chimeric mice that resident microglial activation preceded and predominated over macrophage infiltration in transient focal cerebral ischemia. Furthermore, Gu et al. (2016) showed that the proliferation of resident microglial cells, rather than monocyte infiltration from the blood, was the major contributor to microgliosis in the dorsal horn of the spinal cord after peripheral nerve injury. Similar results were reported by Feng et al. (2019) in an epileptic brain, where resident microglia, but not infiltrated monocytes, were responsible for the hippocampal proliferation of microglial cells after a kainic acid–induced seizure. It has also been reported in recent studies that circulating cells do not infiltrate into the brain parenchyma without irradiation and transplantation or in large quantities (Ajami et al. 2007; Li et al. 2013), which raises the consideration that monocytes may not cross the intact BBB under certain pathological conditions, thus complicating the issue further.

Other experimental approaches have been used to investigate monocyte invasion of peripheral nervous tissue. Using bone marrow chimeric rats, Mueller et al. (2003) identified and quantified hematogenous and resident endoneurial macrophages after sciatic nerve crush injury. The authors found that both resident macrophages and blood-borne monocytes contribute significantly to the total endoneurial macrophage pool during Wallerian degeneration. When the longevity of such macrophages in the PNS of these chimeric animals was further investigated (Müller et al. 2010), a maximal turnover of 75% of hematogenous macrophages was observed, with only a small resident macrophage population. In our studies, we used an alternative method to establish whether resident macrophages respond with increased mitotic activity to noxious stimuli or increased infiltration of monocytes entering the ganglion. Our data revealed that the area of Iba1-immunopositive macrophages significantly increased after dithranol treatment, but this was not in conjunction with an increased number of Ki67-labeled cells. Thus, the lack of mitotic activity eliminated the possibility that resident macrophages were the source of the increased number of Iba1-positive cells. Therefore, our findings indicated that the migration of peripheral macrophages into the trigeminal ganglion was responsible for the increased number of Iba1-positive cells in the ganglion. These data raised the possibility that the microglia/macrophage pools in central and peripheral nervous tissues are maintained differently and that monocyte invasion to the peripheral endoganglionic or endoneurial space is easier than to the parenchyma of the CNS during certain neuropathological or inflammatory events.

Collectively, our novel findings provide further proof that peripheral inflammation, as well as subsequent anti-inflammatory therapy, interferes with the regulation of macrophage activation and could affect neuronal activity both in the periphery and in the CNS (Salter and Beggs 2014; Spangenberg et al. 2016). Further work on the in vivo cellular interactions and mechanisms involved in neuroinflammation and microglia/macrophage proliferation may help to understand the development of neuropathic pain hypersensitivity and neuronal loss.

## Abbreviations

ANOVA: Analysis of variance
BBB: Blood–brain barrier
CaM: Calmodulin
CCR2: C-C chemokine receptor type 2
CD45, CD68: Cluster of differentiation 45 and 68
CNS: Central nervous system
CX3CR1: CX3C chemokine receptor 1 (also known as the fractalkine receptor)
GAPDH: Glyceraldehyde 3-phosphate dehydrogenase
H&E: Hematoxylin and eosin
Iba1: Ionized calcium-binding adaptor molecule 1
IgG: Immunoglobulin G
Ki67: Proliferation marker antigen identified by the monoclonal antibody Ki67 (encoded by the MKI67 gene)
NeuN: Anti-neuronal nuclei protein (hexaribonucleotide-binding protein-3)
PBS: Phosphate-buffered saline
PGP9.5: Pan-neuronal marker protein gene product 9.5 (also known as UCHL-1)
PNS: Peripheral nervous system
RT: Room temperature
SEM: Standard error of the mean
SDS: Sodium dodecyl sulfate
TBS: Tris-buffered saline
UCHL-1: Ubiquitin carboxyl-terminal hydrolase L1 (also known as PGP9.5)

## References

[CR1] Ahmed Z, Shaw G, Sharma VP, Yang C, McGowan E, Dickson DW (2007). Actin-binding proteins coronin-1a and IBA-1 are effective microglial markers for immunohistochemistry. J Histochem Cytochem.

[CR2] Ajami B, Bennett JL, Krieger C, Tetzlaff W, Rossi FM (2007). Local self-renewal can sustain CNS microglia maintenance and function throughout adult life. Nat Neurosci.

[CR3] Aldridge GM, Podrebarac DM, Greenough WT, Weiler IJ (2008). The use of total protein stains as loading controls: an alternative to high-abundance single-protein controls in semi-quantitative immunoblotting. J Neurosci Methods.

[CR4] Beggs S, Salter MW (2007). Stereological and somatotopic analysis of the spinal microglial response to peripheral nerve injury. Brain Behav Immun.

[CR5] Beliczai Z, Varszegi S, Gulyas B, Halldin C, Kasa P, Gulya K (2008). Immunohistoblot analysis on whole human hemispheres from normal and Alzheimer diseased brains. Neurochem Int.

[CR6] Cady RJ, Hirst JJ, Durham PL (2010). Dietary grape seed polyphenols repress neuron and glia activation in trigeminal ganglion and trigeminal nucleus caudalis. Mol Pain.

[CR7] Cuylen S, Blaukopf C, Politi AZ, Müller-Reichert T, Neumann B, Poser I, Ellenberg J, Hyman AA, Gerlich DW (2016). Ki-67 acts as a biological surfactant to disperse mitotic chromosomes. Nature.

[CR8] David S, Kroner A (2011). Repertoire of microglial and macrophage responses after spinal cord injury. Nat Rev Neurosci.

[CR9] Djukic M, Mildner A, Schmidt H, Czesnik D, Brück W, Priller J, Nau R, Prinz M (2006). Circulating monocytes engraft in the brain, differentiate into microglia and contribute to the pathology following meningitis in mice. Brain.

[CR10] Echeverry S, Shi XQ, Rivest S, Zhang J (2011). Peripheral nerve injury alters blood-spinal cord barrier functional and molecular integrity through a selective inflammatory pathway. J Neurosci.

[CR11] Endo Y, Shoji N, Shimada Y, Kasahara E, Iikubo M, Sato T, Sasano T, Ichikawa H (2014). Prednisolone induces microglial activation in the subnucleus caudalis of the rat trigeminal sensory complex. Cell Mol Neurobiol.

[CR12] Feng L, Murugan M, Bosco DB, Liu Y, Peng J, Worrell GA, Wang HL, Ta LE, Richardson JR, Shen Y, Wu LJ (2019). Microglial proliferation and monocyte infiltration contribute to microgliosis following status epilepticus. Glia.

[CR13] Franceschini A, Vilotti S, Ferrari MD, van den Maagdenberg AM, Nistri A, Fabbretti E (2013). TNFα levels and macrophages expression reflect an inflammatory potential of trigeminal ganglia in a mouse model of familial hemiplegic migraine. PLoS One.

[CR14] Gilmore SA, Skinner RD (1979). Intraspinal non-neuronal cellular responses to peripheral nerve injury. Anat Rec.

[CR15] Ginhoux F, Greter M, Leboeuf M, Nandi S, See P, Gokhan S, Mehler MF, Conway SJ, Ng LG, Stanley ER, Samokhvalov IM, Merad M (2010). Fate mapping analysis reveals that adult microglia derive from primitive macrophages. Science.

[CR16] Glenn JA, Sonceau JB, Wynder HJ, Thomas WE (1993). Histochemical evidence for microglia-like macrophages in the rat trigeminal ganglion. J Anat.

[CR17] Greenhalgh AD, David S (2014). Differences in the phagocytic response of microglia and peripheral macrophages after spinal cord injury and its effects on cell death. J Neurosci.

[CR18] Gu N, Peng J, Murugan M, Wang X, Eyo UB, Sun D, Ren Y, DiCicco-Bloom E, Young W, Dong H, Wu LJ (2016). Spinal microgliosis due to resident microglial proliferation is required for pain hypersensitivity after peripheral nerve injury. Cell Rep.

[CR19] Hendriks AG, Keijsers RR, de Jong EM, Seyger MM, van de Kerkhof PC (2012). Combinations of classical time-honoured topicals in plaque psoriasis: a systematic review. J Eur Acad Dermatol Venereol.

[CR20] Hinkerohe D, Smikalla D, Schoebel A, Haghikia A, Zoidl G, Haase CG, Schlegel U, Faustmann PM (2010). Dexamethasone prevents LPS-induced microglial activation and astroglial impairment in an experimental bacterial meningitis co-culture model. Brain Res.

[CR21] Kata D, Földesi I, Feher LZ, Hackler L, Puskas GL, Gulya K (2016). Rosuvastatin enhances anti-inflammatory and inhibits pro-inflammatory functions in cultured microglial cells. Neuroscience.

[CR22] Kata D, Földesi I, Feher LZ, Hackler L, Puskas GL, Gulya K (2017). A novel pleiotropic effect of aspirin: beneficial regulation of pro- and anti-inflammatory mechanisms in microglial cells. Brain Res Bull.

[CR23] Kettenmann H, Hanisch UK, Noda M, Verkhratsky A (2011). Physiology of microglia. Physiol Rev.

[CR24] Kiefer R, Kreutzberg GW (1991). Effects of dexamethasone on microglial activation in vivo: selective downregulation of major histocompatibility complex class II expression in regenerating facial nucleus. J Neuroimmunol.

[CR25] Kreutzberg GW (1996). Microglia: a sensor for pathological events in the CNS. Trends Neurosci.

[CR26] Lange RW, Germolec DR, Foley JF, Luster MI (1998). Antioxidants attenuate anthralin-induced skin inflammation in BALB/c mice: role of specific proinflammatory cytokines. J Leukoc Biol.

[CR27] Leskovar A, Moriarty LJ, Turek JJ, Schoenlein IA, Borgens RB (2000). The macrophage in acute neural injury: changes in cell numbers over time and levels of cytokine production in mammalian central and peripheral nervous systems. J Exp Biol.

[CR28] Li T, Pang S, Yu Y, Wu X, Guo J, Zhang S (2013). Proliferation of parenchymal microglia is the main source of microgliosis after ischaemic stroke. Brain.

[CR29] Lowry OH, Rosebrough NJ, Farr AL, Randall RJ (1951). Protein measurement with the Folin phenol reagent. J Biol Chem.

[CR30] Matsumoto Y, Fujiwara M (1987). Absence of donor-type major histocompatibility complex class I antigen-bearing microglia in the rat central nervous system of radiation bone marrow chimeras. J Neuroimmunol.

[CR31] Menasria R, Canivet C, Piret J, Boivin G (2015). Infiltration pattern of blood monocytes into the central nervous system during experimental herpes simplex virus encephalitis. PLoS One.

[CR32] Mori I, Goshima F, Koshizuka T, Imai Y, Kohsaka S, Koide N, Sugiyama T, Yoshida T, Yokochi T, Kimura Y, Nishiyama Y (2003). Iba1-expressing microglia respond to herpes simplex virus infection in the mouse trigeminal ganglion. Mol Brain Res.

[CR33] Mueller M, Leonhard C, Wacker K, Ringelstein EB, Okabe M, Hickey WF, Kiefer R (2003). Macrophage response to peripheral nerve injury: the quantitative contribution of resident and hematogenous macrophages. Lab Investig.

[CR34] Müller M, Leonhard C, Krauthausen M, Wacker K, Kiefer R (2010). On the longevity of resident endoneurial macrophages in the peripheral nervous system: a study of physiological macrophage turnover in bone marrow chimeric mice. J Peripher Nerv Syst.

[CR35] Orojan I, Szigeti C, Varszegi S, Dobo E, Gulya K (2006). Dithranol abolishes UCH-L1 immunoreactivity in the nerve fibers of the rat orofacial skin. Brain Res.

[CR36] Orojan I, Bakota L, Gulya K (2008). Trans-synaptic regulation of calmodulin gene expression after experimentally induced orofacial inflammation and subsequent corticosteroid treatment in the principal sensory and motor trigeminal nuclei of the rat. Neurochem Int.

[CR37] Prinz M, Priller J, Sisodia SS, Ransohoff RM (2011). Heterogeneity of CNS myeloid cells and their roles in neurodegeneration. Nat Neurosci.

[CR38] Salter MW, Beggs S (2014). Sublime microglia: expanding roles for the guardians of the CNS. Cell.

[CR39] Sawada A, Niiyama Y, Ataka K, Nagaishi K, Yamakage M, Fujimiya M (2014). Suppression of bone marrow-derived microglia in the amygdala improves anxiety-like behavior induced by chronic partial sciatic nerve ligation in mice. Pain.

[CR40] Schilling M, Besselmann M, Leonhard C, Mueller M, Ringelstein EB, Kiefer R (2003). Microglial activation precedes and predominates over macrophage infiltration in transient focal cerebral ischemia: a study in green fluorescent protein transgenic bone marrow chimeric mice. Exp Neurol.

[CR41] Schneider CA, Rasband WS, Eliceiri KW (2012). NIH image to ImageJ: 25 years of image analysis. Nat Methods.

[CR42] Sehgal VN, Verma P, Khurana A (2014). Anthralin/dithranol in dermatology. Int J Dermatol.

[CR43] Spangenberg EE, Lee RJ, Najafi AR, Rice RA, Elmore MR, Blurton-Jones M, West BL, Green KN (2016). Eliminating microglia in Alzheimer’s mice prevents neuronal loss without modulating amyloid-β pathology. Brain.

[CR44] Streit WJ, Semple-Rowland SL, Hurley SD, Miller RC, Popovich PG, Stokes BT (1998). Cytokine mRNA profiles in contused spinal cord and axotomized facial nucleus suggest a beneficial role for inflammation and gliosis. Exp Neurol.

[CR45] Streit WJ, Walter SA, Pennel NA (1999). Reactive microgliosis. Prog Neurobiol.

[CR46] Szabo M, Gulya K (2013). Development of the microglial phenotype in culture. Neurosci.

[CR47] Szabo M, Dulka K, Gulya K (2016). Calmodulin inhibition regulates morphological and functional changes related to the actin cytoskeleton in pure microglial cells. Brain Res Bull.

[CR48] Takahashi K, Watanabe M, Suekawa Y, Ito G, Inubushi T, Hirose N, Murasaki K, Hiyama S, Uchida T, Tanne K (2011). IL-1beta in the trigeminal subnucleus caudalis contributes to extra-territorial allodynia/hyperalgesia following a trigeminal nerve injury. Eur J Pain.

[CR49] Town T, Nikolic V, Tan J (2005). The microglial “activation” continuum: from innate to adaptive responses. J Neuroinflamm.

[CR50] Villa G, Ceruti S, Zanardelli M, Magni G, Jasmin L, Ohara PT, Abbracchio MP (2010). Temporomandibular joint inflammation activates glial and immune cells in both the trigeminal ganglia and in the spinal trigeminal nucleus. Mol Pain.

[CR51] Waite PME, Tracey DJ, Paxinos G (1995). Trigeminal sensory system. The rat nervous system.

[CR52] Wu Y, Wu M, He G, Zhang X, Li W, Gao Y, Li Z, Wang Z, Zhang C (2012). Glyceraldehyde-3-phosphate dehydrogenase: a universal internal control for Western blots in prokaryotic and eukaryotic cells. Anal Biochem.

[CR53] Xu M, Aita M, Chavkin C (2008). Partial infraorbital nerve ligation as a model of trigeminal nerve injury in the mouse: behavioral, neural, and glial reactions. J Pain.

[CR54] Zhang J, Shi XQ, Echeverry S, Mogil JS, De Koninck Y, Rivest S (2007). Expression of CCR2 in both resident and bone marrow-derived microglia plays a critical role in neuropathic pain. J Neurosci.

